# Some aspects of medical education in the former Soviet Union

**DOI:** 10.1590/S1516-31802012000100012

**Published:** 2012-02-13

**Authors:** Sergei Jargin

**Affiliations:** I MD. Associate Professor, Peoples’ Friendship University of Russia, Moscow, Russia.

This topic might be of interest today because of the broadening of international cooperation. The attitude towards academic education in the Soviet Union was complex right from its early days. On the one hand, many young people strove to achieve academic diplomas; on the other hand, the prestige and relative income of educated people started to decrease from the 1950s onwards. One of the reasons for this was overproduction of graduate specialists, together with the low level of knowledge that was on average required. Attendance at lectures was stimulated through administrative measures, but many students neither listened nor wrote down anything, if they were even present at a lecture. For example, biochemistry was regarded by many students as useless, while pharmacology was studied by some of them through textbooks for nursing schools, and it was largely believed that nothing more was really necessary. Nearer to the time of graduation, some students became more diligent in studies within their chosen field. This matter was made additionally complicated because of the limited access to foreign literature, and the uneven quality of Russian-language professional editions,^1^ which resulted in backwardness in some practical fields.

According to my estimates after more than seven years of practicing pathology abroad, the average tumor size in routine surgical specimens (from the stomach, intestine, breast, uterus, prostate, skin, etc.) was at least two to three times larger in Moscow clinics than in provincial hospitals in some Western European countries. This means that early detection of malignancies was less efficient in Russia. Abroad, almost all mastectomy specimens do not include muscle tissue. In Moscow hospitals, modified radical mastectomy (Patey) with removal of the pectoralis minor muscle has been the predominant method over the last 10-15 years. The Halsted operation, with removal of both pectoralis muscles, is also applied. Halsted mastectomy was previously the most prevalent method: it was recommended by Russian-language textbooks of surgery and oncology for all types of breast cancer until the 1990s and even later. Partial gastrectomy for treating duodenal and gastric ulcers was applied abroad much more rarely than in Russia, and the resected volume was less extensive. The use of partial gastrectomy for ulcer treatment was disproportionately high in many institutions until recently,^2^ which could be explained by technical problems, conservatism among surgeons^3^ and limited availability of medical therapy, including in particular, eradication of *Helicobacter pylori*.^2^

So-called administrative factors played a role in this, i.e. endorsement of certain methods by the healthcare authorities, which sometimes favored less individualized methods that were applicable en masse to large contingents of patients. Such practices obviously contributed towards a high negative appendectomy rate^4^ and towards persistence of some outdated methods, like routinely performed diathermocoagulation or cryotherapy on cervical pseudoerosions (cervical ectopy). This practice is at variance with the scientific evidence, which does not support the hypothesis that coagulation of the ectopy provides protection against cervical cancer.^5^

In conclusion, limited availability of foreign professional literature^1^ and partial isolation of Russian medicine from the rest of the world have contributed towards persistence of outdated methods in practice. Therefore, more cooperation with the international community is needed, including temporary practice by Russian physicians and lecturers abroad and by foreign colleagues in Russia.
